# Transplant-Acquired Food Allergy in Children

**DOI:** 10.3390/nu16183201

**Published:** 2024-09-22

**Authors:** Cristiana Indolfi, Angela Klain, Giulio Dinardo, Carolina Grella, Alessandra Perrotta, Simone Colosimo, Fabio Decimo, Michele Miraglia del Giudice

**Affiliations:** Department of Woman, Child and of General and Specialized Surgery, University of Campania Luigi, Vanvitelli, 80138 Naples, Italy; cristianaind@hotmail.com (C.I.); klainangela95@gmail.com (A.K.); alessandraperrotta@icloud.com (A.P.); simone.co345@gmail.com (S.C.); fabio.decimo@unicampania.it (F.D.); michele.miragliadelgiudice@unicampania.it (M.M.d.G.)

**Keywords:** immunosuppressive agents, pediatric transplantation, food hypersensitivity, organ transplantation, intestinal permeability, food allergies, food allergies post-transplant, graft rejection, tacrolimus, cyclosporine

## Abstract

Background: Organ transplantation in children is a vital procedure for those with end-stage organ failure, but it has been linked to the development of post-transplant allergies, especially food allergies. This phenomenon, known as transplant-acquired food allergy (TAFA), is becoming increasingly recognized, though its mechanisms remain under investigation. Pediatric transplant recipients often require lifelong immunosuppressive therapy to prevent graft rejection, which can alter immune function and heighten the risk of allergic reactions. Our review aimed to gather the latest evidence on TAFA. Methods: We conducted a PubMed search from 25 June to 5 July 2024, using specific search terms, identifying 143 articles. After screening, 36 studies were included: 24 retrospective studies, 1 prospective study, 2 cross-sectional researches, and 9 case reports/series. Results: Most studies focused on liver transplants in children. The prevalence of food allergies ranged from 3.3% to 54.3%. Tacrolimus, alongside corticosteroids, was the most commonly used immunosuppressive therapy. In addition to food allergies, some patients developed atopic dermatitis, asthma, and rhinitis. Allergic symptoms typically emerged within a year post-transplant, with common allergens including milk, eggs, fish, nuts, soy, wheat, and shellfish. Both IgE-mediated and non-IgE-mediated reactions were observed, with treatment often involving the removal of offending foods and the use of adrenaline when necessary. Conclusions: Consistent immunological monitoring, such as skin prick tests and IgE level assessments, is essential for early detection and management of allergies in these patients. Understanding the link between transplantation and allergy development is crucial for improving long-term outcomes for pediatric transplant recipients.

## 1. Introduction

**Figure 1:**
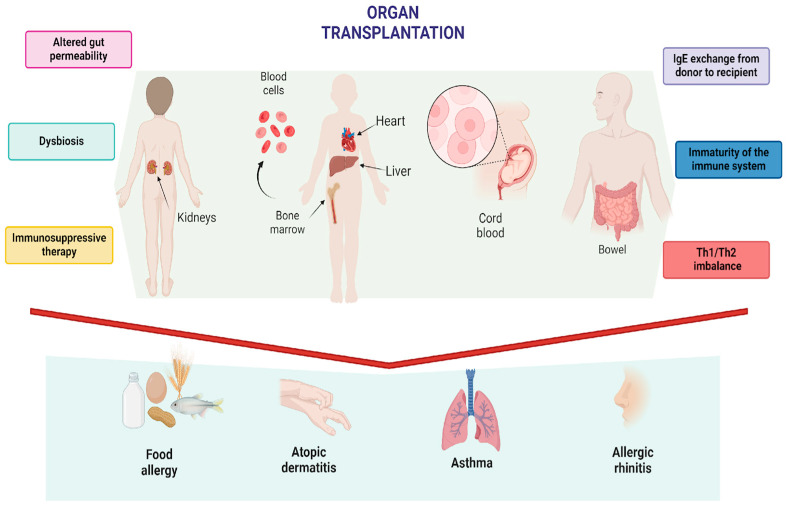
Pathogenetic theories on the development of atopy following organ transplantation. Figure created with BioRender.com.

Food allergies (FAs) have emerged as a significant global health concern, affecting millions of individuals worldwide. The prevalence of FAs has been on the rise, particularly in developed nations, where they now impact an estimated 5–10% of the population [1,2]. These allergies can manifest in a spectrum of clinical symptoms, ranging from mild cutaneous reactions to severe, life-threatening anaphylaxis [3,4]. The increasing prevalence of FAs poses significant challenges for healthcare systems, prompting ongoing research into prevention, diagnosis, and management strategies. Today, there are an increasing number of therapeutic options available, ranging from allergen immunotherapy to biologic treatments, offering hope for better management and potential desensitization in affected individuals [5,6,7]. The correlation between organ transplants and the development of allergies represents an area of growing interest in the field of transplant medicine. Transplants are life-saving procedures used to treat conditions such as organ failure, cancer, and autoimmune diseases [8,9,10]. Some studies report the occurrence of transplant-acquired food allergies (TAFAs) or the recurrence of previously tolerated ones [11,12,13,14]. These allergies occur, particularly, in pediatric patients but have also been documented in adults [15]. A survey highlighted that most sensitizations after a transplant are due to inhalants, particularly dust mites and pollens [16]. The prevalence of new inhalant allergies in transplanted patients can vary between 10% and 20%, depending on the studied populations and the types of transplants considered [17]. Regarding FAs in patients undergoing solid organ transplants, the prevalence is variable and depends on the type of organ transplanted and the patient’s age. Epidemiological studies indicate that FAs are prevalent in pediatric patients undergoing liver transplants, with rates ranging from 5.6% to 38%, depending on the case series considered [18]. TAFAs have also been observed after heart, lung, cord blood, kidney, and intestine transplants, although with a lower frequency compared to liver transplants [14,18,19,20,21,22,23,24,25,26,27]. Among children, the prevalence is significantly higher than in adults, with rates reaching 17% in some studies [18]. Most new FAs develop within the first year after the transplant. Studies indicate that the foods most commonly causing TAFAs are eggs, soy, wheat, and peanuts [15,28]. These allergies have been observed to improve with growth in 69% of cases, with younger children at the time of transplant being more likely to maintain the allergies over the long term [18]. Moreover, some evidence suggests that the transplant may induce the loss of acquired food tolerance, with cases of recurrence of pre-existing FAs or the onset of new allergies after the transplant [11,29]. TAFAs present a variable clinical pattern, ranging from mild skin reactions to potentially life-threatening anaphylaxis [30]. The analysis of the various articles cited in this review clearly shows that the pathogenesis of this phenomenon remains largely unknown. So far, only hypotheses have been proposed, with none receiving definitive confirmation (Figure 1).

### 1.1. Transplant-Related Hypothesis

Considering that the liver is frequently implicated in allergic reactions following transplantation, it has been suggested that liver dysregulation itself could contribute to a loss of tolerance. Indeed, an increase in FAs among children with liver diseases has been documented, highlighting the critical role of hepatic mechanisms in establishing immune tolerance to dietary antigens [20]. This mechanism explains the development of tolerance following years after the liver transplant in patients with TAFAs, due to the restoration of liver function. Most research regarding de novo FAs following liver, bone marrow and bowel transplant support the burden of hematopoietic tissue involved in this phenomenon. In fact, besides the bone marrow, the liver and bowel are organs rich in pluripotent hematopoietic stem cells and resident dendritic cells, which might facilitate sensitization to allergens in the recipient [31,32,33]. Some authors have suggested that the transmission of allergen-specific immunoglobulin E (IgE) antibodies and T lymphocytes from the donor to the recipient during the transplant could play a crucial role [29,34,35]. This can occur if the donor is sensitized to certain allergens such as foods, pollens or drugs. The passive transfer of IgE or lymphocytes may explain most TAFAs occurring in the first months/year after the transplant and the fact that most reactions occur in young children, due to the immaturity of the immune system [36]. The acquisition of tolerance would represent a process related to the maturation of the immune system, similar to that in an adult individual. The reverse scenario, meaning the transplantation of stem cells from a non-allergic donor to an allergic recipient, could also raise the tolerance threshold for previously intolerable foods [37].

### 1.2. Immunosuppressive-Related Hypothesis

Another significant factor is the use of immunosuppressive agents such as tacrolimus (FK506) and cyclosporine A (CsA), which are essential for preventing the rejection of the transplanted organ but can alter the recipient’s immune response, increasing susceptibility to allergies [38,39]. In pediatric transplanted patients treated with tacrolimus and basiliximab, FAs emerged more quickly compared to those treated with other immunosuppressive protocols [40]. De Brunye R. et al. have shown that tacrolimus, in particular, is associated with increased intestinal permeability and an imbalance in type-2 helper T cell (Th2) responses, promoting IgE production and, consequently, allergic reactions [41,42]. This is because immunosuppression aimed at reducing transplant rejection can suppress Th1-mediated inflammatory cytokines, favoring a shift from Th1 to Th2 and increasing the production of IgEs, which can lead to the development of FAs and eosinophilic inflammation. Research conducted by Nahum A et al. has shown that eosinophilic gastrointestinal inflammation (EGI) is common in pediatric transplant patients, with a significantly higher prevalence compared to the general population [43]. Moreover, it has been observed that children with post-liver transplant FAs present a unique cytokine profile characterized by high levels of IL-5 and low levels of IL-10, measured in peripheral blood mononuclear cells, and an increase in serum IgA levels [44]. This increase may be indicative of a disturbance in intestinal immune homeostasis, potentially due to increased intestinal permeability and/or increased IgA production [21]. The study by Mori et al. found that patients who have undergone a liver transplant exhibit elevated levels of natural killer (NK) cells, even higher than those who have undergone a kidney transplant. These patients could be treated with mycophenolate, in addition to tacrolimus, due to its greater inhibition of NK cells, with the expectation of reducing the risk of allergies [22,45]. According to this hypothesis, the phenomenon of FAs following a liver transplant, therefore, seems to derive from a combination of increased intestinal permeability, immunosuppression-induced immune dysregulation, and a predisposition of the recipient.

### 1.3. Microbiome-Related Hypothesis

It is now recognized that the microbiome plays a significant role in the development and prevention of allergies, particularly how intestinal dysbiosis can predispose individuals to the development of FAs. The study by Ellen De Paepe et al. demonstrated that intestinal dysbiosis may precede allergic inflammation in children with IgE-mediated cow’s milk allergy. Researchers used mice sensitized to beta-lactoglobulin and observed changes in microbial metabolites, followed by allergic inflammation. Comparing these results with allergic children, they found that intestinal dysbiosis precedes the development of chronic inflammation, which can then lead to allergic inflammation [46]. Among the causes of intestinal dysbiosis is transplantation, particularly liver transplantation. Lai et al. examined the intestinal microbiome of 37 Chinese adult patients undergoing liver transplantation using 16S rRNA sequencing and demonstrated changes in the bacterial flora after the transplant, with an increase in pathogenic bacteria such as *Enterococcus* and *Klebsiella* and a decrease in beneficial bacteria such as *Bacteroides* and *Bifidobacterium* [47]. Changes in the microbiome were also demonstrated by D’Amico et al. in their prospective study, where they analyzed fecal samples from 91 patients undergoing liver transplantation concerning colonization and infection by carbapenem-resistant *Enterobacterales* (CRE). They found that CRE_I patients showed an immediate and sustained reduction in alpha diversity post-transplant, with a depletion of the gut microbiota structure and a gradual overrepresentation of *Klebsiella* and *Enterococcus* [48]. It is therefore appropriate to emphasize the importance of intestinal dysbiosis in liver transplant recipients not only due to the risk of developing allergies but also of intestinal infections and cholangitis, some of which are caused by bacteria resistant to common antibiotics. 

This review aims to collect and discuss the most recent evidence regarding TAFAs and the underlying physiopathological mechanism to raise consciousness regarding these conditions and possible preventive strategies.

## 2. Methods

A comprehensive electronic search was performed on the PubMed database from 25 June 2024 to 5 July 2024. A subcommittee identified keywords to develop the search strategy for each research question. The research focused on the following PICO terms:

P: Children/Child/Adolescent;

I: Transplant Or Transplantation Or Organ Transplant;

C: Non-Transplanted Children;

O: Food Allergy Or Food Hypersensitivity.

A specific search string was used: ((children or child or adolescent) and (transplant or transplantation or organ transplant)) and (food allergy or food hypersensitivity)). Our inclusion criteria encompassed randomized controlled trials (RCTs), case-control studies, retrospective or prospective studies, clinical trials, case reports, and case series. We excluded systematic reviews, narrative reviews, meta-analyses, studies published solely in non-English languages, and studies lacking free full-text access. Moreover, we added the filter: birth–18 years. Initial study selection was conducted by one author (A.K.), and another author independently verified the accuracy (C.G.). Any discrepancies were resolved through iterative discussion with a third author (C.I.). Furthermore, at least two reviewers (C.I. and G.D.) agreed on the specific data to be extracted from the included studies. Eligibility was determined based on the evaluation of titles and abstracts against the inclusion criteria. Studies meeting these criteria underwent full-text review to determine final inclusion. 

## 3. Results

**Figure 2:**
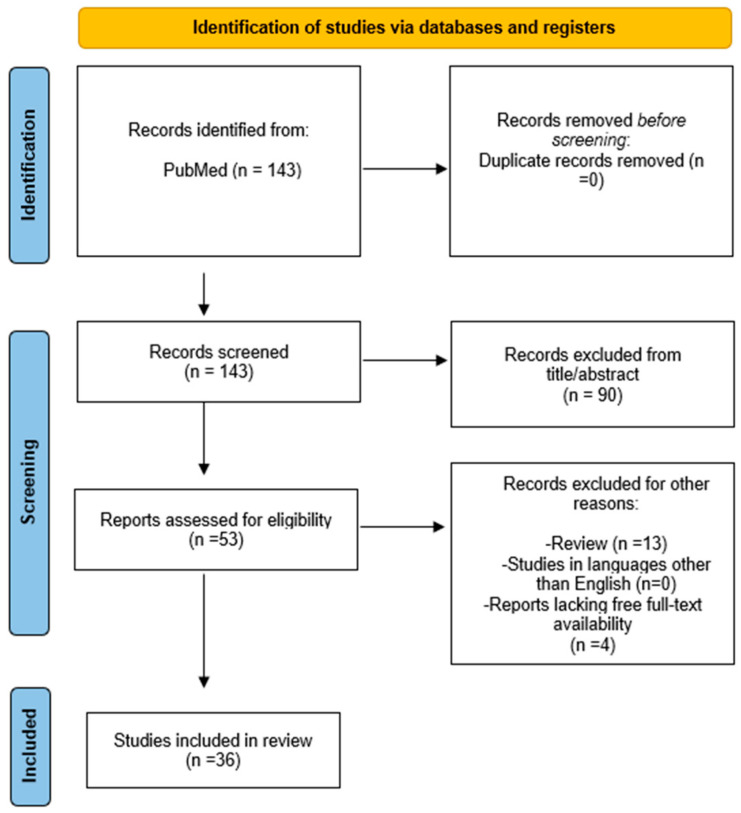
PRISMA flow diagram.

The study selection process involved identifying 143 records from PubMed. After screening, 90 records were excluded based on title and abstract. An additional 17 records were excluded for other reasons, including 13 reviews and 4 reports lacking free full-text availability. Ultimately, 53 reports were assessed for eligibility, and 36 studies were included in the final review [12,13,14,19,20,23,24,25,26,27,31,41,49,50,51,52,53,54,55,56,57,58,59,60,61,62,63,64,65,66,67,68,69,70,71,72] (Figure 2). 

Of the included studies, 24 articles were retrospective studies, 1 was a prospective study [52], 2 were cross-sectional studies [14,49] and 9 were case reports/series [31,56,57,61,68,69,70,71,72]. All the articles included pediatric populations who have undergone organ transplantation. Most of the studies examined the development of atopy following different types of organ transplantation. The most involved organ transplant was the liver: 33 studies involved post-liver transplanted patients, 6 kidney [12,14,19,25,27,41], 3 heart [14,25,56], 3 bowel [14,25,65], 2 bone marrow [68,69], and 2 cord blood transplants [23,57]. Regarding immunosuppressive therapy, steroids were used in all the studies, as they are included in all transplantation protocols; tacrolimus was used in 36 studies, cyclosporine in 19 studies, sirolimus in 3 studies, azathioprine in 2 studies and mycophenolate mofetil in 7 studies. In all seven studies, mycophenolate mofetil was used, and it is interesting to note that there was no significant difference in the development of allergies between patients treated with mycophenolate mofetil and those who received other immunosuppressive therapies. In six out of seven studies, it was observed that, on average, around 20–30% of these patients later developed food allergies, asthma, and atopic dermatitis. In one out of seven studies, 3.3% of patients developed food allergies, although it was not determined whether asthma and atopic dermatitis also developed. Although our primary search focused on the development of TAFAs, we also found results related to the development of other allergies. In 14 studies, the development of atopic dermatitis was observed [12,13,14,20,24,25,41,49,51,55,58,62,65,67], in 15 studies asthma [12,13,14,20,22,25,49,51,54,58,59,62,64,66,67], and in 9 articles both asthma and rhinitis were reported [12,13,14,20,25,41,51,58,62]. Regarding the timing of the development of allergic symptoms, we observed that in 17 studies, symptoms appeared on average within a year after the transplant; in 9 studies, within 2 years; in 2 studies, within 3 years; and in 1 study, within 6 years. The foods most frequently linked to FAs were milk, eggs, fish, nuts, soy, wheat, and shellfish. Additionally, studies have identified other allergens such as fruits (banana, kiwi, apple) [12,13,63,66], sesame [19,55,61], potatoes [13], pork and chicken [23], as well as lentils, beef, horse, and lamb [60,63]. Both IgE-mediated FA reactions, documented by clinical symptoms (immediate reactions) and epicutaneous and laboratory findings (skin prick test and specific IgEs), and non-IgE-mediated forms have been observed. In all studies, the treatment approach involved removing the offending food(s) and, when required, administering adrenaline. We also encountered several intriguing and distinctive case reports, including one where a peanut allergy resolved after bone marrow transplantation for primary immunodeficiency [69], another describing necrotizing enterocolitis due to cow’s milk allergy in a pediatric patient following liver transplantation [70], and a case of tacrolimus-associated eosinophilic gastroenterocolitis in pediatric liver transplant recipients [72]. Additionally, there was a case series on the persistence of FAs in patients with dedicator of cytokinesis 8 protein (DOCK8) deficiency who underwent hematopoietic stem cell transplantation [68], as well as a report on subtotal villous atrophy linked to food protein sensitivity in three pediatric liver transplant recipients receiving tacrolimus [71].

**Figure 3:**
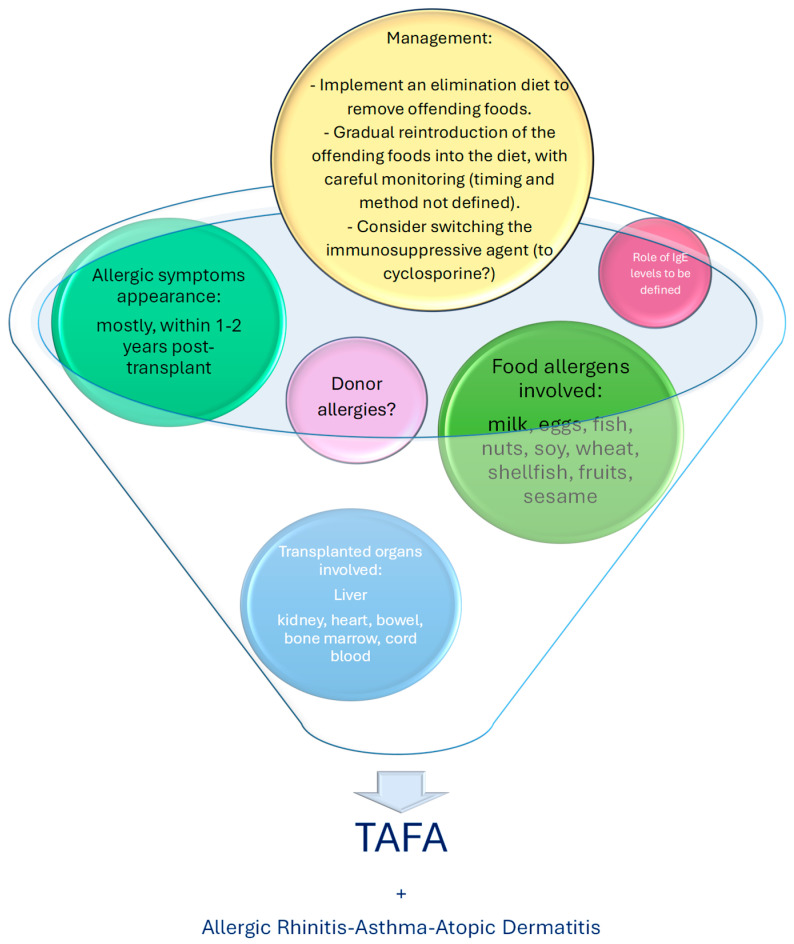
This figure presents a graphical analysis of the key findings related to the development of TAFA in pediatric patients. Certain aspects, including donor characteristics, the role of IgE level measurement, and case management, remain important areas that require further clarification.

Most studies have shown that the development of FAs occurs, generally, after one year post-transplant but before two years [12,13,19,22,24,27,44,51,58,59,60,65]; fewer studies have shown an earlier development of TAFAs, within one year post-transplant [23,25,41,52,54,55,62,64,67]. In the majority of studies, IgE levels varied from very low to over 1000 IU/L [19,23,41,55,60,63,66,67]. No significant link was identified between IgE levels and the severity of the clinical presentation. In Ozbek et al.’s research, IgE levels were measured both before the transplant and at 3, 6 and 12 months post-transplant. While post-transplant IgE levels were higher than those measured prior, they never exceeded 300 IU/L. One study observed that liver transplant patients had higher IgE levels compared to kidney transplant recipients, but this difference was not statistically significant (*p* = 0.08) [41]. We found limited data available concerning donor characteristics. Most research focuses on distinguishing between organs from deceased donors and those from living donors, whether related or not. In many studies, the donors were living-related [13,26,27,50,52,67], whereas in others, transplanted organs were derived from deceased donors [14,22,51,58,65]. One study, in particular, concentrated on orthotopic liver transplants [24]. Only three studies analyzed the atopic profile of donors. One study found that none of the donors had a history of FAs and all the donors tested negative for food-specific IgE or skin prick tests, including those connected to children who later developed FAs [52]; another study revealed that 11 donors had a history of atopy, but none had a history of FAs [25]. In the final study, some donors had allergic rhinitis, but only one had a history of shellfish allergy [53]. Three studies demonstrated that donor allergy was not a risk factor for the development of allergies in the recipients [44,50,64]. Only a few studies have discussed the management of TAFAs. In three studies, therapy was limited to the elimination of offending foods [27,58,67], but no significant success rates were reported. In seven studies, allergen reintroduction was attempted, with mixed results: some were successful [23,50,53,54], while others were not [25,41,51]. In eight studies, modifications to immunosuppressive therapy were implemented [14,24,50,51,52,53,63,65]. In Barış et al.’s study, treatment was adjusted to a combination of tacrolimus and everolimus in two patients, sirolimus in two others, and cyclosporine in another two patients [50]. In two other studies, immunosuppressive therapy was switched from tacrolimus to cyclosporine A: in one study, this did not impact allergic reactions [52], while in the other one, the successful reintroduction of food allergens occurred only in patients who were switched to cyclosporine [63]. In the research by Haflidadottir et al., the introduction of mycophenolate mofetil in transplantation programs led to a reduction in FAs after liver transplants in children. Moreover, treatment with mycophenolate mofetil at one and two years post-liver transplant, in combination with tacrolimus, was associated with a lower incidence of FAs and sensitization [24]. In one study, immunosuppressive therapy was reduced or discontinued in 11% of patients with FAs [51]. All the studies that included data regarding the follow-up of transplanted patients had at least 1 year of follow-up [13,14,19,24,25,27,41,51,52,53,54,59,62,63,64,65] (Table 1) (Figure 3).

## 4. Discussion

FAs are a widespread health issue, affecting up to 5–10% of the global population. These allergies can lead to a variety of health problems, ranging from mild discomfort to severe, life-threatening reactions, with an increasing number of treatment options now available [1,73]. The broad range of symptoms and severity levels seen in FAs are reflected in the diverse clinical manifestations triggered by different foods and the varying threshold doses required to elicit a reaction [74]. In the context of transplantation, the development of FAs, referred to as TAFAs, introduces additional layers of complexity. These allergies not only pose significant health risks but also impose substantial emotional, social, and economic burdens on affected individuals and their families [75]. Understanding the mechanisms behind the onset of TAFAs, and examining how they relate to the type of transplant, the immunosuppressive therapy used, and the patient’s gut microbiome, could help us prevent FAs from developing post-transplant. Our research highlights the significant prevalence and complexity of TAFAs in pediatric patients, particularly in those who have undergone liver transplantation. In a recent systematic review on the development of TAFAs following liver transplantation in pediatric patients, which included 40 articles, the results showed that 15% of transplanted patients develop de novo FAs, mostly within the first two years post-surgery, with younger age and tacrolimus immunosuppression being key risk factors. Multiple FAs and anaphylaxis are common (15% of incidence), and most patients do not outgrow their symptoms. Switching from tacrolimus to cyclosporine or combining tacrolimus with mycophenolate has shown potential for improving or resolving allergies in severe or difficult cases [76]. Our findings reveal that up to 33% of pediatric liver transplant recipients developed TAFAs, which aligns with the liver’s critical role in immune tolerance and its unique immunological properties [12]. The data also indicate a notable incidence of other allergic manifestations, such as asthma (17%) and rhinitis (19%), suggesting a broader spectrum of immune dysregulation in these patients. One of the most critical observations is the timing of allergy onset post-transplantation, with symptoms emerging between 1.5 to 3.6 years after the procedure [12], with a higher risk of developing allergic symptoms in the first year of life compared to later years [13]. This period appears to be a crucial window for the development of allergic sensitization, potentially driven by the interaction between the immunosuppressive regimen and the immature immune system in pediatric patients. Specifically, the use of tacrolimus, a calcineurin inhibitor frequently employed in post-transplant immunosuppression, has been associated with increased intestinal permeability and a shift towards Th2-dominated immune responses, both of which are known to facilitate allergic sensitization [21,42]. Interestingly, we observed that switching the immunosuppressant to cyclosporine A was associated with improved outcomes, with higher success rates in the improvement or resolution of allergies, with no report of graft rejection [50,60,63]. However, the data are still very limited, and further randomized controlled studies are needed. FAs were most commonly linked to common allergens such as milk, eggs, fish, nuts, and shellfish, and both IgE-mediated and non-IgE-mediated allergic reactions were observed. Interestingly, although IgE levels varied significantly across studies, no clear link was found between these levels and the severity of allergic symptoms. In some studies, improvement or normalization of specific IgE levels and skin prick test results were used to determine the timing for oral food challenges (OFC) and the reintroduction of trigger foods into the diet [50,52,61]. Donor allergy profiles were examined in a few studies, particularly regarding their allergy profile. Three studies did not find any correlation between the donor’s allergy profile and the development of TAFAs in the recipient [51,53,64]. Overall, while these findings are valuable, the data remain limited, and more extensive trials are needed to better understand the role of donor characteristics and the management of allergies in transplant patients.

The microbiome’s role in TAFA development also emerged as a significant factor in our analysis. Alterations in gut microbiota, characterized by a reduction in beneficial bacteria and an increase in pathogenic strains, were observed in liver transplant recipients. These changes are likely to contribute to intestinal dysbiosis, which has been implicated in the pathogenesis of allergic diseases. The correlation between microbiome alterations and the onset of TAFAs underscores the need for further research into gut microbiota modulation as a potential therapeutic strategy [46,47,48]. Our data also revealed a variable prevalence of allergies across different types of organ transplants and immunosuppressive regimens. For instance, liver transplant recipients exhibited a higher incidence of TAFAs compared to those receiving other organs, which may be attributable to the liver’s unique immune environment [52,53]. The association between tacrolimus and a higher rate of FAs compared to other immunosuppressants, such as corticosteroids, suggests that specific agents may have more profound effects on immune modulation and allergic risk. This finding highlights the necessity of tailoring immunosuppressive protocols to minimize the risk of developing new allergies [60,67]. The clinical manifestations of TAFAs in our cohort ranged from mild reactions to severe, life-threatening anaphylaxis [50]. This variability in clinical presentation emphasizes the importance of vigilant monitoring and prompt management of allergic reactions in pediatric transplant recipients. Moreover, the persistence of FAs in some patients, particularly those transplanted at a younger age, raises concerns about long-term outcomes and quality of life [75].

In clinical practice, donor selection typically relies on standardized criteria to prevent infections and organ rejection, and specific allergological evaluations are rarely conducted. However, based on the potential risk of allergy transmission, incorporating a more comprehensive donor screening, including allergen profiles and IgE antibody testing, could be valuable in the future, particularly for individuals with a reported history of FAs. Moreover, to enhance the quality of data related to post-transplant allergies, it is necessary to implement more effective data collection methods. One option could be the creation of a centralized registry for transplant patients that includes specific information on post-transplant-acquired allergies. Such a registry would facilitate long-term patient monitoring and allow for more detailed data collection on FAs and their progression. These concerns further underscore the need for ongoing research into more effective prevention and treatment strategies. 

### Study Limitations and Strengths

Our review has several limitations that should be noted. Firstly, we relied exclusively on a single database, PubMed, which may have constrained the breadth of our research and potentially led to the exclusion of pertinent studies available in other databases. While PubMed is a leading database and includes a substantial number of indexed journals, its use alone may limit the comprehensiveness of our review. Additionally, it is important to note that our review was not a systematic analysis, which further impacts the thoroughness of our search. Secondly, the search string used in our review was designed with relatively simple terms and was not highly selective. This approach was intended to facilitate the retrieval of a broad range of results. However, this broad search strategy may have introduced some bias in identifying relevant articles. A more refined search string could have potentially captured a more targeted selection of studies, thus influencing the outcomes and conclusions of our review. Another key limitation is the potential underreporting of cases, as many instances of post-transplant FAs may not have been adequately documented. Furthermore, the absence of detailed information regarding donor-derived cases poses an additional challenge to fully understanding the phenomenon. This issue is further compounded by the limited availability of specific data in current registries. Lastly, our review did not include an in-depth comparative analysis of the methodologies and outcomes reported in the studies we reviewed. This limitation arises from the diversity in study methodologies, such as variations in study design, sample sizes, data collection methods, and statistical analyses. The differences in how outcomes were measured and reported across the studies further complicated our ability to perform a comparative analysis. This lack of comparative depth might affect the interpretation and integration of the findings. Despite these limitations, our review offers a valuable and inclusive summary of the existing research on TAFAs in children. It provides a consolidated view of the current state of knowledge, highlighting an issue that remains relatively underrecognized, despite its frequency. The impact of this problem on the quality of life for patients, who are already vulnerable and fragile, is significant. By bringing attention to this issue, we aim to enhance awareness and understanding, which could lead to improved management and support for affected children and their families.

## 5. Conclusions

In conclusion, while organ transplantation remains a life-saving intervention, the associated risk of developing FAs, particularly in pediatric liver transplant recipients, is a significant concern that warrants further investigation. Understanding the interplay between immunosuppressive therapy, organ-specific immune properties, microbiome alterations, and patient-specific factors is essential to developing targeted interventions that can mitigate this risk. Our review highlights the growing concern of TAFAs, particularly in pediatric liver transplant recipients. Preventing TAFAs requires a comprehensive and multidisciplinary approach. Additionally, current donor selection focuses on preventing infections and organ rejection. Implementing enhanced allergological evaluations and centralized data collection on post-transplant allergies could improve patient outcomes and provide valuable insights into allergy progression. Optimizing immunosuppressive therapy is essential; this includes customizing medication protocols and exploring immunomodulatory drugs to lower the chances of new allergies. Dietary management post-transplant can also play a significant role, with a gradual introduction of foods and avoidance of common allergens to allow the recipient’s immune system to adapt. Education for patients and their families is critical, focusing on recognizing allergic reactions and using emergency medications such as auto-injectors. Regular immunological monitoring, including skin prick tests, IgE levels and allergy symptoms, helps in early detection and intervention. Furthermore, advancing research through longitudinal studies and biomarker development will refine risk assessment and prevention techniques. The modulation of the gut microbiome via probiotics, prebiotics, or fecal microbiota transplantation could offer novel preventive avenues. Implementing these strategies could significantly reduce TAFA incidence and improve long-term outcomes for transplant patients, highlighting the importance of an integrated, personalized approach to transplant care. Future research should focus on elucidating the underlying mechanisms of TAFA and exploring the role of immunosuppression and the microbiome in this phenomenon. Additionally, understanding the interplay between donor and recipient immune systems can pave the way for innovative therapeutic approaches. By addressing these areas, we can better support the long-term health and quality of life of pediatric transplant patients.

## Figures and Tables

**Table 1:** This table provides an overview of all the studies included in the review and their respective characteristics. For practical reasons, case reports and case series are not featured in the table. ND: no data.

Reference	Study Population	Age at Transplant	Study Type	Transplant	Immunosuppressive Therapy	Food Allergy	Atopic Dermatitis	Asthma/ Rhinitis	Time Point to a Diagnosis of TAFA after Transplantation	Donor Characteristics	Serum IgE	Management	Duration of Follow-Up
Roberts et al. (2023) [12]	232 pediatric and adult patients	Median age: Liver: 1.6 (0.75–4) years Kidney: 9.2 (3.7–12.3) years	Retrospective	Liver, Kidney	Tacrolimus, Mycophenolate, Corticosteroids	Yes (33% pediatric liver)	Yes (73% pediatric liver)	Yes (19% rhinitis, 17% asthma pediatric liver)	1.8 years (1.3–4.0)	Type of donor (living vs. deceased) not significantly associated with TAFA development	ND	ND	ND
Almaas et al. (2019) [49]	59 liver transplant recipients, 56 with chronic liver disease (control)	Median age: 0.8 years (0.6–5.2)	Cross-sectional	Liver	Tacrolimus, Mycophenolate, Prednisolone	Yes (39% in transplanted children)	Yes (41% in transplanted children)	Yes (24% asthma in transplanted children)	1.5 years (0.5–3.0)	A total of 57 patients received organs from deceased donors, while 2 were recipients of living-donor transplants	Median: 32 IU/mL (9–165)	ND	ND
Catal et al. (2015) [13]	49 pediatric liver transplant recipients	Median age: 5 (0.3–16.5) years	Retrospective	Liver	Tacrolimus, Cyclosporine	Yes (12.2%)	Yes (6.1%)	Yes (12.2% asthma, 4.1% rhinitis)	The reaction occurred within the first year post-transplant in 5 out of 6 patients.	Cadaveric organ: 13 (26.5) Living-related: 33 (67.4) Living non-related: 3 (6.1)	12 patients (24.5%) had serum total IgE levels > 100 IU/mL	ND	16 months (1–47)
Marcus et al. (2018) [14]	273 pediatric solid-organ transplant recipients	Median age: Liver: 1.7 years (0.8–6.9) Heart: 1.2 years (0.4–9.2) Kidney: 10.8 years (6.3–15.5) Multivisceral: 1.2 years (0.9–1.6) Total: 2.9 years (0.7–10.3)	Cross-sectional cohort	Liver, Heart, Kidney, Multivisceral	Tacrolimus, Steroids	Yes (25.3%)	Yes (16.1%)	Yes (10.3% asthma, 5.5% rhinitis)	ND	A total of 47 (42.3%) liver and 30 (57.7%) kidney recipients were transplanted with living-donor organs. Organ type (living donor vs. cadaveric), donor/recipient blood type and compatibility were not associated with TAFA development.	ND	Of the 92 children, 10 (11%) were managed conservatively, 65 (71%) received standard medical treatment, and 17 (18%) had their immunosuppressive therapy adjusted after medical treatment failure. Following this change, 9 patients showed improvement, 7 achieved full resolution, 13 remained unaffected, and 4 worsened, including 2 fatalities.	Liver: 3.2 years (1.6–5.8) Heart: 4.5 years (2.8–6.8) Kidney: 2.4 years (1.2–6.0) Multivisceral: 4.8 years (1.6–5.5) Total 3.6 years (1.7–6.3)
Barış et al. (2019) [50]	236 pediatric liver transplant recipients	The mean age: 7.92 ± 2.64 years (range, 4.8–15.6)	Retrospective	Liver	Tacrolimus, Steroids	Yes, 8%	ND	ND	ND	A total of 18 patients underwent living-related liver transplants, with the donor being the mother in 11 cases, the father in 6 cases, and a secondary relative in 2 cases. One patient, who received a deceased-donor liver transplant at 1 year of age due to acute liver failure, required a retransplant at age 5.5 from his mother because of chronic rejection.	Mean level: 350 ± 411 IU/mL	All food allergens were successfully reintroduced in 7 patients (36.8%), while 8 patients with multiple FAs were able to reintroduce only some foods. Milk was reintroduced after an average of 22.8 ± 14.5 months (range 7–54 months), egg after 15.8 ± 8 months (range 7–29 months), and wheat after 30.5 ± 35.7 months (range 11–84 months). The immunosuppressive regimen was changed to a combination of tacrolimus and everolimus in 2 patients, to sirolimus in another 2, and to cyclosporine in 2 patients.	4.76 ± 3.97 years
Wisniewski et al. (2012) [51]	352 pediatric liver transplant recipients	Median age: 0.9 (0.6–2.0) years	Retrospective	Liver	Tacrolimus, Cyclosporine, Prednisone	Yes (8.5%)	Yes (43%)	Yes (20% asthma, 20% rhinitis)	Median 1.0 ( 0.5–8.2) years post-liver transplantation	The median donor age was 32 years (range 1–71) in the FA group. Female donors (both living and deceased) provided 40% of the organs for FA recipients compared to 50% for controls (*p* = 0.48). CMV+ donors made up 44% of the FA livers and 56% of the controls (*p* = 0.43). 12 were living-related transplants, and 17 cadaveric ones. No statistically significant associations were found regarding donor age or sex or type of transplantation.	ND	A total of 14 children remained on avoidance diets, 10 followed unrestricted diets, and 6 were lost to follow-up. Immune suppression was lowered or discontinued in 11% of FAs.	Median 10 (7.3–4.0) years
Levy et al. (2009) [19]	297 pediatric transplant recipients	Mean age: Kidney: 10.8 years (range: 2–18). Liver: 5.5 years (range: 0.5–17.5)	Retrospective	Liver, Kidney	Tacrolimus, Cyclosporine, Prednisone	Yes (4 out of 65 liver recipients)	ND	ND	1.5–6 years	ND	IgE levels were: 224,155 and 3900 UI/mL.	Tacrolimus therapy was switched to cyclosporin A in 2 patients, but there was no change in their food-induced allergic reactions. All patients were advised to eliminate the allergenic foods from their diets, which led to symptom resolution. No efforts were made to reintroduce the allergenic foods during the follow-up period	2–4 years
Ozbek et al. (2009) [52]	28 pediatric liver transplant recipients	Mean age: 4.96 +/− 0.76 years	Prospective	Liver	Tacrolimus, Cyclosporine, Sirolimus	Yes (21%)	ND	ND	The time between transplantation and onset of FA was 3, 6 (in 2 patients), 11, 12, and 20 months (mean 9.7 months).	None of the donors had a history of FAs. Food-specific IgE tests or skin prick tests were negative in all 28 donors, including those linked to children who later developed FAs. All the patients received a living-related donor organ transplantation.	Before transplantation: 44.88 UI/mL After 3 months PT: 116.63 UI/mL After 6 months PT: 98.40 UI/mL After 12 months PT: 276.63 UI/mL	Patients who developed FAs after liver transplantation were placed on elimination diets, resolving their symptoms, with no reintroduction of allergenic foods. In some cases, immunosuppressive therapy was switched (tacrolimus-> cyclosporine A), but this did not impact the allergic reactions.	Mean follow-up time: 25.4 months (range 12–40 months)
Sinitkul et al. (2018) [53]	46 pediatric liver transplant recipients	Median age: 19.1 months (15.3–34.2)	Retrospective	Liver	Tacrolimus, Corticosteroids	Yes (54.3%)	ND	ND	12.2 months (6.2–21.3 months)	Some donors had allergic rhinitis, but only one had a history of shellfish allergy. However, this did not significantly influence the development of FAs in recipients	The levels of IgE ranged from very low values just above positivity to values exceeding 100.	Patients who developed FAs were placed on elimination diets, avoiding allergenic foods. Reintroduction of foods was attempted after 3 years of elimination, with about 19% of patients developing tolerance to at least one allergen. No changes in the immunosuppressive therapy were associated with the resolution of FAs.	Median 59.5 months (57.2, 92.8)
Sakashita et al. (2012) [23]	14 pediatric cord blood transplant recipients	Mean age: 1.6 ± 1.3 years in symptomatic patients, 5.6 ± 4.5 years in asymptomatic ones	Retrospective	Cord Blood (CB)	Tacrolimus, Cyclosporine, Methotrexate, Methylprednisolone	Yes (5 out of 14)	No	No	3 to 6 months after CBT	Four patients received transplants of unrelated CB cells; one was related.	Total IgE levels reached more than 3000 UI/mL)	Eliminating the suspected food(s) resolved the symptoms in all 5 patients.	ND
Ozbek et al. (2015) [54]	28 pediatric liver transplant recipients	Mean age: 10.16 years	Retrospective	Liver	Tacrolimus, Cyclosporine, Sirolimus	Yes (21%)	ND	Yes (asthma in one patient)	Mean 9.66 months (33 +/− 19 months)	ND	ND	The systematic elimination of allergens from the diet was maintained in all cases. An oral challenge with each allergen was conducted. The allergens were successfully reintroduced in 4 children within 7 to 38 months of starting the elimination diet.	5 years
De Bruyne et al. (2013) [41]	49 liver, 21 renal transplant recipients	Median (and range) age: Liver: 22 Months (3 weeks–16 years) Renal: 8.9 (2–15) years	Retrospective	Liver, Renal	Tacrolimus, Cyclosporine, Mycophenolate mofetil, Steroids	Yes (26.5% liver, 0% renal)	Yes (In the non-food-allergic group, 7 of 36 atopic dermatitis)	Yes (In the non-food-allergic group, 2 of 36 children have asthma or allergic rhinitis)	Median 8 months post-transplant (range 1–48 months)	Liver: Living-related (7) Liver: Cadaveric organ (42) Renal: Living-related (2) Renal: Cadaveric organ (19)	Mean IgE levels 5.84 kU/L. Total IgE was higher in liver transplanted patients (24.4 kU/L (0–9930) versus 7 kU/L (0–157)) (*p* = 0.08).	All patients with FAs systematically eliminated the identified allergens from their diets. In 4 patients, the allergens were successfully reintroduced within 7 to 38 months after starting the elimination diet	Liver: 67 (19–230) Months Renal: 74 (10–166) Months
Mitsui et al. (2017) [55]	206 pediatric liver transplant recipients	Median age: 9 months (6.0–14.3)	Retrospective	Liver	Tacrolimus, Steroids	Yes (20.4%)	Yes	ND	Median 3 months post-transplant (range 1–8 months)	Median age: 33.0 years (30.0–38.0)	Ig-E mediated FA: Median 129 (15.6–1048) Non-IgE-mediated FA: median 52.7 (22.4–131)	ND	ND
Haflidadottir et al. (2022) [24]	107 pediatric liver transplant recipients	Median age: 1.9 years (0.7–8.3 years)	Retrospective	Liver	Tacrolimus, Mycophenolate Mofetil (MMF), Prednisolone	Yes (22%)	Yes	Yes (asthma)	Median 1.6 (0.6–3.3) years.	A total of 124 patients underwent orthotopic liver transplantation. Three patients underwent living-donor liver transplantation; the rest of the patients received split or whole liver from deceased donor.	ND	The introduction of mycophenolate mofetil in the transplantation program led to a reduction in FAs following liver transplantation in children. Additionally, treatment with mycophenolate mofetil at 1 and 2 years post-liver transplantation, alongside tacrolimus, was linked to decreased FAs and food sensitization.	Median 7.6 years (2.5–13.6 years)
Frischmeyer-Guerrerio et al. (2008) [25]	25 pediatric solid organ transplant recipients	Median age: 8.7 months (5.8–13.3 months)	Retrospective	Liver, Small Bowel, Heart, Kidney	Tacrolimus, Mycophenolate Mofetil, Corticosteroids	Yes	Yes	Yes (*n* = 11 rhinitis; *n* = 4 asthma)	Median 6.0 months (4.4–10.4 months)	Eleven donors had a history of atopy, but none had a history of FAs. Twelve transplants were living-related liver.	ND	Elimination diet. Of 25 patients, 3 followed unrestricted diets at the time of last follow-up	Median 2.4 years (1.4- 4.7 years) in the clinic and 6.1 years (3.9–7.5 years) by telephone
Noble et al. (2011) [58]	78 pediatric liver transplant recipients	Range 0.1–17.3 years	Retrospective	Live	Tacrolimus, Cyclosporine	Yes (20%)	Yes	Yes (*n* = 1 rhinitis; *n* = 5 asthma)	Range from 2 months to 6 years	A total of 78 children received liver transplants from 85 cadaveric donors	ND	All children were treated with the appropriate medications or allergen avoidance measures, including dietary restrictions, and for those with eosinophilic esophagitis (EE), oral steroids and swallowed topical steroid sprays were used.	ND
Brown et al. (2012) [20]	50 pediatric liver transplant recipients	Median age: 12.1 (7.9–20.6) months	Retrospective	Liver	Tacrolimus, Cyclosporine, Mycophenolate Mofetil	Yes (20%)	Yes	Yes (23% asthma and allergic rhinitis)	ND	ND	Median 15.0 (4.0–105.5) UI/mL	ND	ND
Wasuwanich et al. (2021) [59]	98 pediatric liver transplant recipients	Median age: 3.3 years (1.1–9.3)	Retrospective	Liver	Tacrolimus	Yes (7%)	ND	Yes (asthma)	Median time: 1.9 years (0.8–3.5 years), while the median time to diagnose eosinophilic colitis was 0.5 years (0.4–1.4 years)	A total of 28 (29%) of the 96 children had live-donor liver transplantation	ND	ND	At least one year after transplantation
Öztürk et al. (2019) [26]	60 pediatric liver transplant recipients	Mean age: 6.1 years (3 months to 17 years)	Retrospective	Liver	Tacrolimus, Mycophenolate Mofetil, Steroids	Yes (3.3%)	ND	ND	ND	Thirty-nine patients (65%) received livers from living donors, while 21 patients (35%) received livers from deceased donors.	ND	ND	ND
Lebel et al. (2014) [60]	154 pediatric liver transplant recipients	Range: one month to 19 yr	Retrospective	Liver	Tacrolimus, Cyclosporine	Yes (17% Tacrolimus, 3% Cyclosporine)	ND	ND	Median 25 months post-transplant (range 6–94 months)	ND	Mean levels 1082 kU/L	ND	ND
Shroff et al. (2012) [62]	176 pediatric liver transplant recipients	Median age: 16 (3–127; IQR, 7–30) months. Mean age: 26.6 months	Retrospective	Liver	Tacrolimus, Cyclosporine	Yes (40%)	Yes (56%)	Yes (Allergic rhinitis: 64%, asthma 44%)	Median 11.5 (6–28) months post-transplantation Mean: 27.1 months post-transplantation	ND	ND	ND	Median 63 (17–127; IQR, 42–110) months. Mean: 79.0 months
Lykavieris et al. (2003) [63]	121 pediatric liver transplant recipients	Mean age 1.32 years	Retrospective	Liver	Tacrolimus	Yes (10%)	ND	ND	ND	ND	Mean levels 2454 kIU/L	In addition to eliminating food allergens, 8 children were transitioned from tacrolimus to cyclosporine, while the tacrolimus dosage was reduced in 4. Successful reintroduction of food allergens occurred only in those who were switched to cyclosporine.	Mean: 3.75 years; (range 2.8–4.2 years)
Saalman et al. (2010) [27]	39 pediatric liver, 38 pediatric kidney transplant recipients	Median age: 22 months (range 1 month–16 years)	Retrospective	Liver, Kidney	Tacrolimus, Cyclosporine, Azathioprine, Prednisolone	Yes	Long-standing oral lesions including angioedema (7 patients)	ND	Median 21 months (range 6 months to 4.5 years) post-transplant for angioedema	5 living donors, 3 cadavers	ND	Eliminations diet	Mean 6.84 years
Lee et al. (2013) [64]	93 pediatric liver transplant recipients	Median age: 11 months (8–34 months)	Retrospective	Liver	Tacrolimus	Yes (37.6%)	ND	Asthma (3.2%)	Median 5 months post-transplant (IR 2.3–9.5 months)	Donor allergy was not found to be a risk factor for the development of allergy in the recipient (HR 95% CI: 1.271 (0.307–5.271) *p*-value: 0.741)	ND	The management ranged from simple observation to strict antigen restriction or an elemental diet, depending on the patients’ age, clinical symptoms, and feeding methods	Median follow-up: 70 months (41–90 months)
Kehar et al. (2020) [65]	8 pediatric liver/multivisceral transplant recipients	Median age: 1 year (0.5–2.4 years)	Retrospective	Liver (7), Multivisceral (1)	Tacrolimus switched to Sirolimus	Yes (2 out of 8)	Yes (2 out of 8)	ND	Median: 1.3 (0.25–8) years after transplantation	Of the 7 isolated liver transplants, 3 (43%) received an allograft from a living donor, while 4 (57%) received one from a deceased donor	ND	Elimination diet. Eight recipients who underwent either liver (*n* = 7) or multivisceral transplants (*n* = 1) experienced severe, treatment-resistant PTAID (Post-transplant allergy or immune-mediated disease) and were transitioned from tacrolimus to sirolimus.	Median follow-up of 5 years
Granot et al. (2006) [66]	30 pediatric liver transplant recipients	Mean age: 10.6 years (1.9–21 years)	Retrospective	Liver	Tacrolimus, Cyclosporine, Prednisone	Yes (13.3%)	ND	Yes (asthma in 1 patient)	ND	ND	Five patients were < 3 years of age and IgE levels ranged from 54 to 111 IU/mL (mean: 83), Five patients were > or =9 years and IgE levels ranged from 134 to 1606 IU/mL (mean: 557)	ND	ND
Arikan et al. (2003) [67]	50 pediatric liver transplant recipients	Mean age: 9 years (13–5 years)	Retrospective	Liver	Tacrolimus, Cyclosporine, Prednisone	Yes (4%)	Yes	Yes (asthma in 1 patient)	Mean 6.3 months (range 4–9 months)	1 cadaveric donor, 2 living-related donors	Mean 1020 IU/L (range 400–1800 IU/L)	Symptoms resolved with appropriate elimination diets.	ND

## Data Availability

No new data were created or analyzed in this study.
